# Coordination of insulin and Notch pathway activities by microRNA miR-305 mediates adaptive homeostasis in the intestinal stem cells of the *Drosophila* gut

**DOI:** 10.1101/gad.241588.114

**Published:** 2014-11-01

**Authors:** David Foronda, Ruifen Weng, Pushpa Verma, Ya-Wen Chen, Stephen M. Cohen

**Affiliations:** 1 Institute of Molecular and Cell Biology, Singapore 138673, Singapore;; 2 Department of Biological Sciences, National University of Singapore, Singapore 117543, Singapore

**Keywords:** stem cell, microRNA, insulin, Notch

## Abstract

Homeostasis of the intestine is maintained by dynamic regulation of a pool of intestinal stem cells. The balance between stem cell self-renewal and differentiation is regulated by the Notch and insulin signaling pathways. Dependence on the insulin pathway places the stem cell pool under nutritional control, allowing gut homeostasis to adapt to environmental conditions. Here we present evidence that miR-305 is required for adaptive homeostasis of the gut. miR-305 regulates the Notch and insulin pathways in the intestinal stem cells. Notably, miR-305 expression in the stem cells is itself under nutritional control via the insulin pathway. This link places regulation of Notch pathway activity under nutritional control. These findings provide a mechanism through which the insulin pathway controls the balance between stem cell self-renewal and differentiation that is required for adaptive homeostasis in the gut in response to changing environmental conditions.

## Abstract (precis)

Homeostasis of the intestine is maintained by dynamic regulation of a pool of intestinal stem cells. The balance between stem cell self-renewal and differentiation is regulated by the Notch and insulin signaling pathways. Foronda et al. show that miR-305 regulates the Notch and insulin pathways in the intestinal stem cells. miR-305 expression in the stem cells is under nutritional control via the insulin pathway.

Tissue homeostasis depends on populations of self-renewing stem cells. Dynamic control of the stem cell population is critical in high-turnover tissues such as the intestinal epithelium. This is particularly important in the gut, where environmental conditions, including nutritional status and tissue damage, can influence stem cell turnover rates (Amcheslavsky et al. 2009; O’Brien et al. 2011). Stem cell dysfunction is linked to aging of the gut (Biteau et al. 2010; Guo et al. 2014).

*Drosophila* intestinal stem cells (ISCs) divide to produce more ISCs and a nonamplifying transitional cell called the enteroblast (EB) (Micchelli and Perrimon 2006; Ohlstein and Spradling 2006). ISC divisions were initially described as symmetric (producing two ISC daughter cells) or asymmetric (producing an ISC and an EB daughter cell) (Ohlstein and Spradling 2007; O’Brien et al. 2011). The proportion of symmetric and asymmetric outcomes is nutritionally regulated, and this depends on insulin pathway activity in the ISCs (O’Brien et al. 2011). Using two-color clonal analysis to label both daughter cells of ISC divisions, de Navascues et al. (2012) reported three possible outcomes: symmetric divisions producing two ISCs, divisions producing one ISC and one EB daughter cell, and divisions producing no ISCs in which both progeny differentiated. The study showed that the balance between these outcomes, inferred from the ratio of ISCs to EB cells, depends on the level of Notch activity.

The differentiation of the ISC to the other cell types depends largely on the activity of the Notch signaling pathway (Micchelli and Perrimon 2006; Ohlstein and Spradling 2006, 2007). The Notch ligand Delta (Dl) is expressed in the ISCs and activates Notch to promote differentiation of the EB cells. ISCs also express the negative regulator Hairless, which contributes to keeping Notch activity low. Notch pathway activation is reflected in the EB cells by expression of downstream effectors, including Suppressor of Hairless [Su(H)]. Correct interpretation of these signals confers identity to both ISCs and EB cells (Bardin et al. 2010). Notch signaling acts subsequently to determine whether EBs differentiate into secretory enteroendocrine (EE) cells or the larger polyploid enterocytes (ECs), which provide absorptive function, with higher Notch activity promoting the EC fate (Ohlstein and Spradling 2007; Perdigoto et al. 2011; Kapuria et al. 2012).

The insulin/IGF-like signaling (IIS) pathway plays important roles in several aspects of stem cell self-renewal and differentiation. IIS activity is required for ISC division (Amcheslavsky et al. 2009; Biteau et al. 2010; Choi et al. 2011; O’Brien et al. 2011). Differentiation of EB cells into EC cells requires activity of the IIS pathway, although differentiation into EE cells does not (Choi et al. 2011). Interestingly, the proportion of asymmetric ISC divisions producing two different daughter cells versus symmetric divisions producing two ISC daughter cells is influenced by activity of the IIS pathway and is nutritionally regulated (O’Brien et al. 2011). Localized production of dILP3 in midgut muscle influences symmetric versus asymmetric ISC division. During asymmetric division, IIS activity in the EB cell appears to contribute to the separation of the EB daughter cell from the ISC, which is required to allow the ISC to continue proliferating (Choi et al. 2011). However, the fundamental mechanism by which IIS activity controls the symmetric versus asymmetric division remains unclear.

MicroRNAs (miRNAs) have been linked to regulatory feedback and feed-forward mechanisms, which suggests that they may serve as regulators of cellular homeostasis (Herranz and Cohen 2010; Ebert and Sharp 2012). A growing body of evidence indicates that miRNAs play an essential role in stem cells, where cellular homeostasis is crucial for self-renewal and differentiation. Some miRNAs contribute to stem cell maintenance through negatively regulating the expression of genes involved in differentiation (Gangaraju and Lin 2009; Hattangadi et al. 2011; Yi and Fuchs 2012; Shyh-Chang and Daley 2013). In *Drosophila*, genetic analysis has linked miRNAs to regulation of stem cell maintenance and proliferation. *bantam* miRNA has been implicated in the maintenance of ovarian stem cells (Shcherbata et al. 2007). *miR-124* activity is required to support proliferation of neuroblasts in the larval brain by limiting expression of Anachronism (Weng and Cohen 2012). Target sites for *miR-275* and *miR-306* limit the expression of the differentiation factor Bam in male germline stem cells (Eun et al. 2013).

Here, we report on the role of the *miR-305* miRNA in controlling the balance between ISC self-renewal and differentiation. *miR-305* acts on both the Notch and insulin signaling pathways in the ISCs. Deletion of *miR-305* in a targeted knockout mutant results in elevated IIS activity in the ISCs, leading to an expansion of the ISC population at the expense of differentiation. This mimics the normal adaptive response to a nutrient-rich environment (O’Brien et al. 2011) as well as the effects of age-related gut dysplasia (Biteau et al. 2010; Guo et al. 2014). However, even under normal nutritional conditions, loss of the *miR-305* regulatory network leads to premature dysplasia and early death. Intriguingly, *miR-305* expression is itself nutritionally regulated. We propose that regulation of Notch activity as a consequence of IIS-regulated miR-305 expression is responsible for the balance between stem cell pool expansion and differentiation. Thus, *miR-305* is a key element of the adaptive homeostasis mechanism that links ISC population size to nutritional status.

## Results

### Predicted miR-305 targets in ISCs

**Figure 1.**
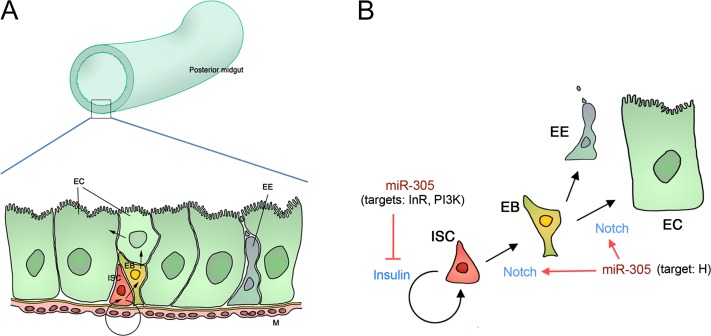
miR-305 targets in ISC self -renewal and differentiation. (*A*) Schematic representation of the intestinal epithelium. EB, EC, and EE cells. (*B*) Predicted *miR-305* targets in signaling pathways involved in ISC self-renewal and differentiation.

Computational miRNA target prediction can generate hypotheses on the functions of specific miRNAs. We made use of Targetscan (http://www.targetscan.org) and the EMBL target prediction tool (Stark et al. 2005) to identify miRNAs targeting signal transduction pathways implicated in stem cell self-renewal and differentiation. We observed that *miR-305* has predicted targets in the insulin and Notch signaling pathways and set out to test whether *miR-305* has a function in ISCs (Fig. 1A,B).

### The miR-275–305 cluster in the control of stem cell number and proliferation

**Figure 2.**
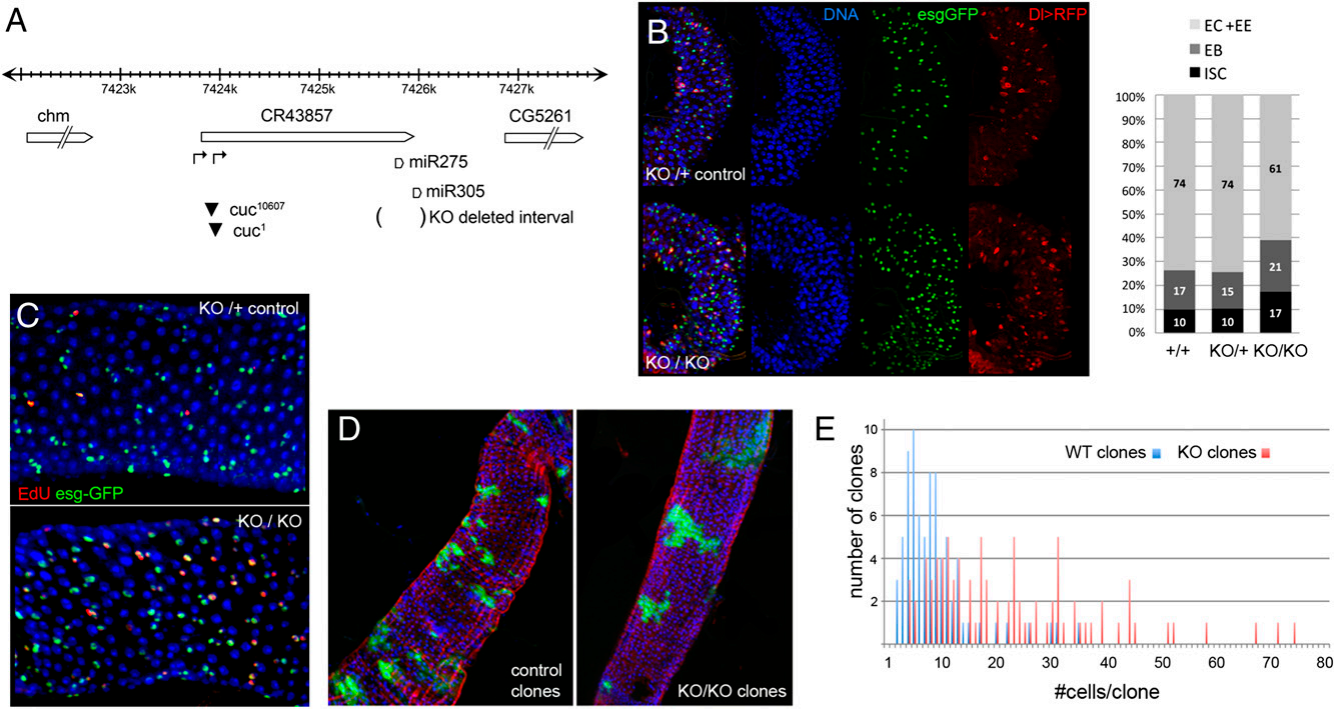
The miR-275/miR-305 cluster controls ISC proliferation. (*A*) *miR-275* and *miR-305* miRNAs are located near the 3′ end of the noncoding transcript CR43857 in the interval between the protein-coding genes *chm* and *CG5261*. All three genes are transcribed in the same orientation. Black triangles indicate P-element insertion sites. Arrows indicate predicted transcription start sites. The interval deleted in the KO allele is indicated by parentheses. The genomic region containing the miRNAs was replaced by a mini-white cassette flanked by LoxP sites (details are in Supplemental Fig. S1A). miR-275 and miR-305 miRNAs were reduced to low levels in animals carrying the P-element insertion alleles *cuc*^*1*^ and *cuc*^*10607*^ in *trans* to the KO allele (Supplemental Fig. S1B). The *cuc* P-element insertions appear to be alleles of *miR-275–305*. Expression of CG5261 was unaffected in the *miR-275–305* deletion mutant, monitored by quantitative RT–PCR (qRT–PCR). (*B*) Posterior midguts were collected from 7-d-old adults and labeled to visualize ISCs (Dl-Gal4>UAS-RFP; red). esg-GFP labels both ISCs and EB cells (green). Nuclei were labeled with DAPI (blue). (*Top* panel) KO/+ control. (*Bottom* panel) KO/KO mutant. (Histogram) Dl-positive and esg-positive cells were counted from seven midgut samples of each genotype, represented as average percent of total cells. ANOVA: *P* = 0.008 comparing the number of Dl-Gal4 cells in KO/KO versus control; *P* = 0.024 comparing esg-Gal4 cells in KO/KO versus control. Persistence of Dl-Gal4-driven RFP expression might lead us to underestimate the proportion of esg-GFP-positive cells that are EBs but would not affect the comparison between genotypes. (*C*) EdU incorporation in KO/+ control and KO/KO mutant midguts. esg-Gal4 was used to label ISCs and EB cells (green). Anti-EdU (red) labels cells that underwent DNA synthesis during the 30-min exposure to EdU. (*D*,*E*) MARCM clones in the midgut labeled with GFP. (*D*, *left*) Control clones. (*Right*) KO/KO mutant clones. (*E*) Mutant clones contained more cells on average.

The *miR-275* and *miR-305* miRNAs are located in close proximity in a region of ∼4 kb between two protein-coding loci (Fig. 2A). The two miRNAs appear to be products of the long noncoding transcript CR43857. The 5′ end of this transcript maps to a hot spot for P-element insertions (http://www.flybase.org) in an area predicted to contain transcription start sites (Ryazansky et al. 2011). We prepared a deletion of the *miR-275-305* cluster by homologous recombination, in which both miRNAs were replaced by a mini-white marker flanked by LoxP sites. Details of the targeting construct and the structure of the mutated locus are shown in Supplemental Figure S1A (for brevity, the targeted knockout allele is referred to as KO). miRNA PCR was used to confirm the absence of the mature miR-275 and miR-305 miRNAs in KO/KO animals and KO/Df(2L)BSC189 animals [Df(2L)BSC189 removes ∼500 kb of DNA, including the *miR-275-305* cluster] (Supplemental Fig. S1B). Survival to adulthood was reduced in the KO/KO homozygous mutant combination as well as in the KO/Df and KO/*cuc*^*1*^ transheterozygous combinations (Supplemental Fig. S1C). The KO/KO, KO/Df, and KO/*cuc*^*1*^ mutants also shared an extra bristle phenotype (Supplemental Fig. S1D). The life span of adult mutant flies was short compared with controls (Supplemental Fig. S1E).

Short adult life span can result from defects in the maintenance of ISCs (Biteau et al. 2010). To examine the stem cell population, we made use of reporters for Dl (an ISC-specific marker) (Ohlstein and Spradling 2007) and *escargot* (*esg*), which labels ISCs and EB cells (Micchelli and Perrimon 2006). We observed an increase in the number of Dl-Gal4-expressing cells and esg-GFP-expressing cells in the adult midgut of *miR-275–305* cluster mutants (KO/Df) compared with the heterozygous KO/+ controls (Fig. 2B). Dl-Gal4-expressing ISCs comprised 17% of cells in the mutant versus 10% in the +/+ and KO/+ controls (ANOVA: *P* = 0.008). EBs (esg-GFP cells that did not express Dl-Gal4) comprised 21% of cells in the mutant versus 15% and 17% in the controls (ANOVA: *P* = 0.02). To ask whether the increase in ISC number was due to elevated proliferation, we measured EdU incorporation. In *miR-275–305* cluster mutants, 37% of esg-GFP-expressing cells incorporated EdU following a half-hour pulse, compared with 8% in the KO/+ control animals (*P* = 0.002, Mann Whitney test) (Fig. 2C). Thus, the increase in ISC and EB populations appears to reflect an increased rate of ISC proliferation. Clones of homozygous *miR-275–305* mutant cells grew larger than control clones, producing a larger number of differentiated progeny (Fig. 2D,E), consistent with the effects of elevated IIS activity on ISC proliferation (Amcheslavsky et al. 2009; Biteau et al. 2010).

### miR-305 acts in ISCs

**Figure 3.**
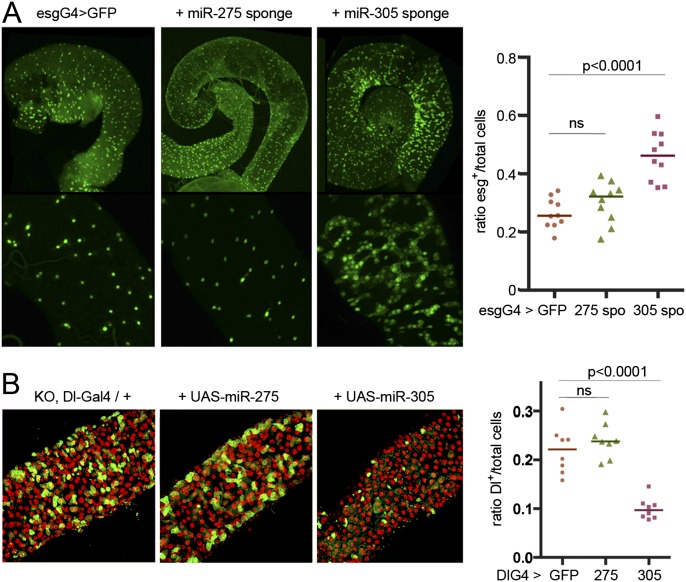
miRNA sponges distinguish between *miR-275* and *miR-305* function. (*A*) esg-Gal4 was used to direct UAS-GFP alone or together with UAS sponge transgenes that deplete miR-275 or miR-305. Depletion of miR-305 increased the number of esg-Gal4-expressing ISCs and EB cells. ANOVA: *P* = 0.4 comparing control with miR-275 sponge and *P* < 0.0001 comparing control with miR-305 sponge. (*B*) Dl-Gal4 was used to express UAS-miR-275 or UAS-miR-305 in ISCs in the KO/KO mutant background. Dl-Gal4>UAS-GFP cells (green). DAPI (red). ANOVA: *P* = 0.5 comparing control with UAS-miR-275; *P* < 0.0001 comparing control with UAS-miR-305.

The *miR-275–305* cluster mutant removes both miRNAs. To assess their individual contributions to the ISC/EB phenotype, we generated miRNA sponges (Loya et al. 2009). For each sponge, 10 miRNA target sites were introduced in the 3′ untranslated region (UTR) of a UAS-dsRed transgene. We made use of esg-Gal4 to direct expression of the UAS-sponge transgenes in ISCs and EB cells. Expression of the UAS-miR-275 sponge had little or no effect on the number of esg-Gal4-positive cells (Fig. 3A), whereas expression of the UAS-miR-305 sponge recapitulated the mutant phenotype, showing a significant increase (Fig. 3A). As a second approach, we made use of Dl-Gal4 to direct expression of each miRNA alone in the *miR-275–305* cluster mutant background. Expression of miR-305 in Dl-Gal4-expressing cells effectively suppressed the increase in ISC number, whereas expression of miR-275 had no effect (Fig. 3B). Together, these observations suggest that loss of miR-305 is primarily responsible for the ISC/EB phenotype observed in the *miR-275–305* cluster mutant.

**Figure 4.**
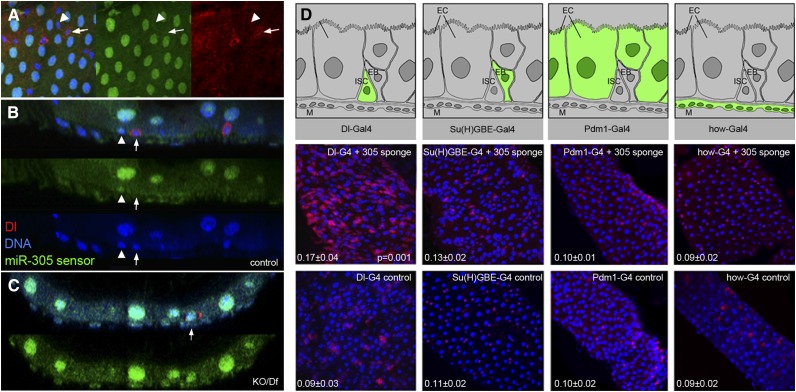
*miR-305* acts in ISCs. (*A–C*) Images of posterior midguts expressing a miR-305 sensor transgene (green). Samples were labeled with anti-Dl to visualize ISCs (red) and with DAPI (blue). (*A*) Normal control, surface view. ISCs and EB cells are diploid and have small nuclei. (Arrows) A Dl-expressing ISC; (arrowheads) adjacent EBs. miR-305 sensor GFP levels were lower in the nuclei of the small cells. This difference disappeared in the miR-305 mutant background (Supplemental Fig. S2). (*B*) Optical cross-section showing an adjacent pair of basally located ISCs and EB cells. miR-305 sensor GFP levels were similar to background GFP levels in the ISCs but were detectable above background in the EB cell. (*C*) miR-305 sensor GFP levels in a 5-d-old KO/Df mutant midgut. The arrowhead indicates a Dl-expressing ISC. The level of GFP expression is comparable in the ISCs and other cells in the *miR-275–305* KO/Df mutant combination. Note the dysplastic appearance of the gut, with mispositioning of the normally basally located ISCs and evidence of Dl expression in partially differentiated cells (partially endoreplicated), features that are more typically found in older flies (see also Supplemental Fig. S2). (*D*, *top* row) Summary of the cell type specificity of the Gal4 drivers. (*Middle* row) Gal4-driven expression of the UAS-miR-305 sponge transgene to selectively deplete miR-305 in ISCs and EB, EC, and EB cells. ISCs were labeled with anti-Dl (red). (*Bottom* row) Gal4 driver controls without the UAS-miR-305 sponge. The ratio of Dl^+^/total cells is shown in the *bottom left* corner for each genotype (average ± standard deviation [SD] from counts of eight midguts). The difference between miR-305 sponge-expressing and control was significant for Dl-Gal4 (*P* = 0.0011, Mann Whitney test). The differences were not significant with the other Gal4 drivers (see Supplemental Fig. S3).

To visualize *miR-305* activity in the ISC lineage, we made use of a miR-305 sensor transgene that contained two perfect sites for miR-305 inserted into the 3′ UTR of a ubiquitously expressed tubulin promoter-GFP transgene. Down-regulation of this type of sensor in miRNA-expressing cells leads to low GFP activity (Brennecke et al. 2003). GFP was strongest in the large polyploid EC cells, indicating low *miR-305* activity. GFP levels were low in the smaller diploid cells, including the Dl-expressing ISCs and the EB cells (Fig. 4A). The miR-305 sensor showed background levels of GFP in the basally located Dl-expressing ISCs (Fig. 4B). GFP was low but detectable in adjacent EB cells (Fig. 4B). GFP levels were high in all cells of the midgut in the KO/Df mutant (*P* = 0.0002, comparing GFP expression in control vs. mutant ISCs) (Fig. 4C; quantitative data is provided in Supplemental Fig. S2). These observations suggest that miR-305 activity is highest in the ISCs and begins to decline in EB cells, perhaps as they begin to differentiate.

To further explore the site of *miR-305* action, we made use of a panel of Gal4 drivers that permit expression of the miRNA sponge transgene in the different cell types of the ISC lineage (Micchelli and Perrimon 2006; Jiang et al. 2009; Zeng et al. 2010). The ratio of Dl-expressing ISCs to total midgut cells was significantly increased when the miR-305 sponge was expressed in the ISCs under Dl-Gal4 control (*P* = 0.001) (Fig. 4D). Expression of the miR-305 sponge in the EB cells under Su(H)GBE-Gal4 control had a limited effect, but the difference was not statistically significant (Supplemental Fig. S3). Expression of the sponge in the EC cells with Pdm1-Gal4 or in the smooth muscle surrounding the gut with how-Gal4 also had no effect on ISC number. These observations provide evidence that miR-305 is expressed and required in the ISCs.

The Notch pathway has been shown to act at two stages in the process of ISC differentiation. The ratio of ISCs to EB cells depends on the level of Notch activity (de Navascues et al. 2012). Notch signaling acts subsequently to determine whether EBs differentiate into secretory EE cells or the larger polyploid ECs, which provide absorptive function, with higher Notch activity promoting EC fate (Ohlstein and Spradling 2007; Perdigoto et al. 2011; Kapuria et al. 2012). We therefore asked whether the balance between production of EE and EC cells was affected in the *miR-305* mutant background. The number of Prospero-expressing EE cells increased significantly in the mutant (*P* < 0.0001, Mann Whitney test) (Supplemental Fig. S4). This is consistent with the expectation that elevated Hairless activity in the mutant EB cells would lower Notch activity, favoring EE differentiation. A shift in the proportion of cells that become diploid EE cells versus polyploidy EC cells may contribute to the reduced size of the mutant gut.

### miR-305 targets the insulin and Notch pathways

**Figure 5.**
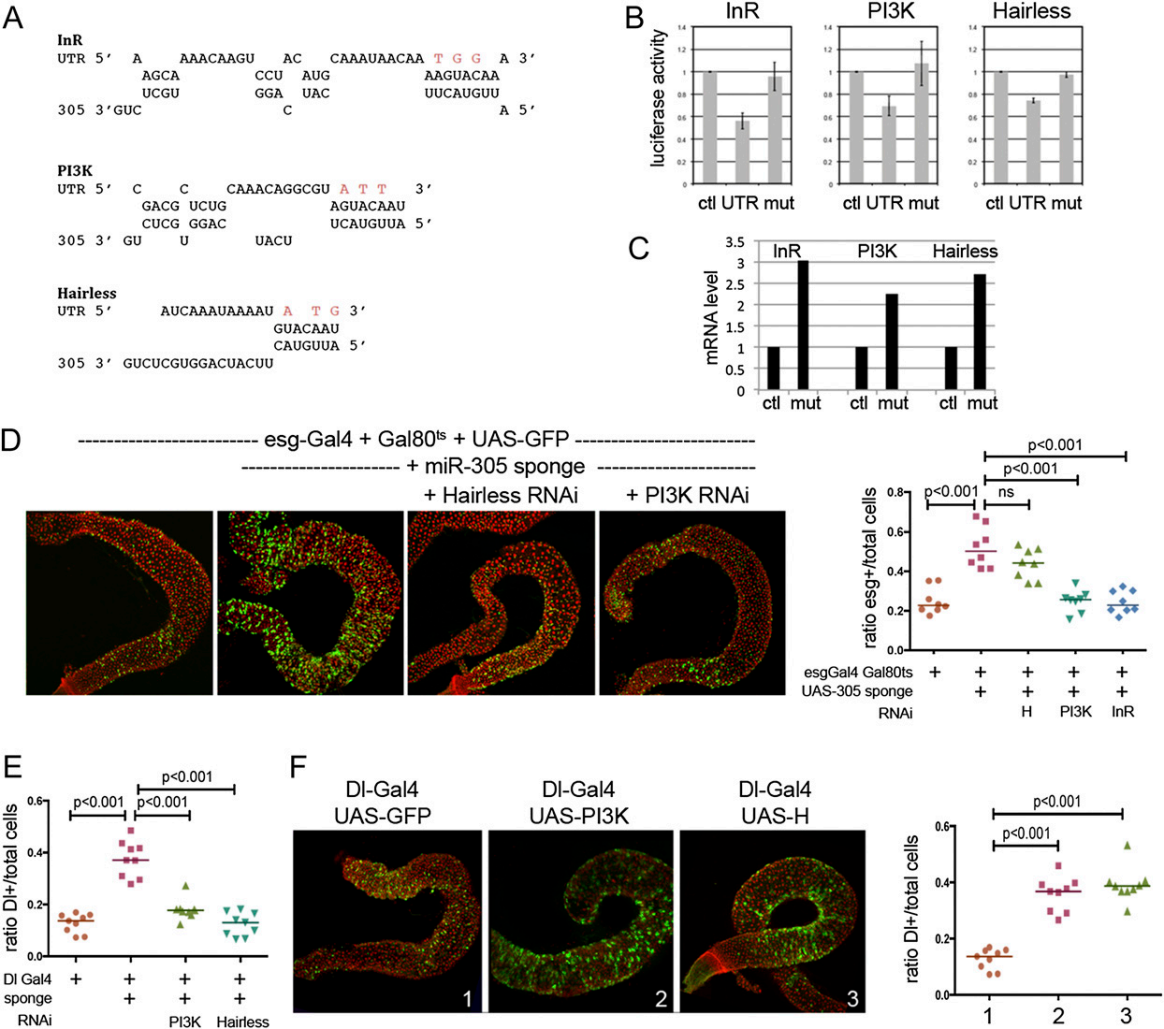
*miR-305* acts via regulation of insulin and Notch pathways in ISCs. (*A*) Predicted target sites for miR-305 in the 3′ UTRs of the mRNAs encoding InR, PI3K, and Hairless. Residues changed in the target site mutant UTR reporters are shown in red. (*B*) Effect of miR-305 on luciferase reporters containing the 3′ UTRs of the indicated transcripts. (Ctl) Control luciferase reporter with an SV40 3′ UTR; (UTR) intact 3′ UTR reporters; (Mut) target site-mutated version of the UTR reporter. In all cases, cells were transfected to express miR-305 under the control of the tubulin promoter. Data are the average of four experiments ± SD. *P* < 0.01 comparing InR, PI3K, or Hairless UTRs with the control UTR; *P* < 0.025 comparing intact and mutant PI3K UTRs; *P* < 0.01 for InR and Hairless (Student’s *t*-test, two-tailed, unequal variance). (*C*) ISC RNA was TU-tagged by expression of UPRT under Dl-Gal4 in the adult midgut. Dl-Gal4 was combined with Gal80^ts^. Animals were reared at 18°C to keep Gal4 inactive and shifted to 29°C at day 5 and aged for 2 d before TU incorporation. InR, PI3K, and Hairless transcript levels were measured by qRT–PCR. Additional replicates are provided in Supplemental Figure S5. (*D*) esg-Gal4 was used to direct transgene expression in the ISCs and EB cells. esg-Gal4 was combined with Gal80^ts^. Animals were reared at 18°C to keep Gal4 inactive and shifted to 29°C at day 2 and aged for 5 d. esg-Gal4-expressing cells (green). (Red) Nuclei labeled with DAPI. (Scatter plot) Ratio of esg-Gal4 cells to total cells. Data were analyzed by ANOVA. (*E*) Dl-Gal was used to direct transgene expression in the ISCs. Dl-Gal4 was combined with Gal80^ts^. Animals were reared at 18°C to keep Gal4 inactive and shifted to 29°C at day 2 and aged for 5 d. The ratio of Dl-Gal4 cells to total cells is shown. Data were analyzed by ANOVA. (*F*) Dl-Gal was used to direct transgene expression in the ISCs. (Panel *1*) Dl-Gal4 + UAS-GFP. (Panel *2*) Dl-Gal4 + UAS-GFP + UAS-PI3K. (Panel *3*) Dl-Gal4 + UAS-GFP + UAS-Hairless. Dl-Gal4-expressing ISCs (green). Nuclei were labeled with DAPI (red). (Scatter plot) Ratio of Dl-Gal4-expressing to total cells. Data were analyzed by ANOVA.

Target sites were predicted for miR-305 in the 3′ UTR of the transcripts encoding insulin receptor (InR) and phosphatidyl-inositol-3-kinase (PI3K92E), a key element in insulin pathway signal transduction (Fig. 5A). Luciferase reporter constructs were prepared containing the 3′ UTRs of these transcripts. Expression of miR-305 in S2 cells led to a reduction of luciferase activity (Fig. 5B). Down-regulation was abrogated by mutating the seed region of the predicted targets sites for InR and PI3K (Fig. 5A,B). Thus, InR and PI3K appear to be targets of miR-305 activity. miR-305 was also predicted to target Hairless, which acts as a negative regulator of Notch activity (Brou et al. 1994; Schweisguth and Posakony 1994). Expression of miR-305 reduced expression of a Hairless 3′ UTR luciferase reporter, and this effect was lost upon mutation of the seed region (Fig. 5A,B).

To test whether these targets were misregulated in the ISCs in the mutant, we measured target mRNA levels by quantitative real-time PCR on RNA from ISCs that was labeled using the TU-tagging method (Miller et al. 2009). The method is based on cell type-specific expression of the uracil phosphoribosyltransferase (UPRT) enzyme, which permits incorporation of a 4-thiouracil (4-TU) base into newly synthesized mRNA. Dl-Gal4 was used to direct UPRT expression in ISCs. *InR*, *PI3K*, and *Hairless* transcripts were up-regulated in KO/KO mutant ISCs (Fig. 5C; Supplemental Fig. S5).

We used two genetic tests to ask whether misregulation of these targets in the ISCs contributed to the defects observed the *miR-275–305* cluster mutant. First, esg-Gal4 was used to express the UAS-miR-305 sponge transgene to deplete miR-305 in the ISCs and EB cells. The animals also carried Gal80^ts^ to limit activation of Gal4 to the adult, following a shift to the permissive temperature. miR-305 depletion increased the number of ISCs/EB cells, phenocopying the mutant (Fig. 5D). Depletion of InR or PI3K in this genetic background effectively suppressed the increase in ISC/EB cell number caused by miR-305 depletion (Fig. 5D). Similar results were obtained using Dl-Gal4 to drive transgene expression in ISCs: Depletion of PI3K suppressed the effects of miR-305 depletion (Fig. 5E). Depletion of Hairless did not cause a statistically significant reduction in ISC/EB cell numbers when expressed under esg-Gal4 control (Fig. 5D) but did so when expressed under Dl-Gal4 control (Fig. 5E). Second, we used Dl-Gal4 to overexpress the targets in an otherwise wild-type background. Overexpression of Hairless or PI3K increased the number of Dl-expressing ISCs (Fig. 5F). These results indicate that misregulation of *InR*, *PI3K*, and *Hairless* each contributed to the increase in ISC/EB cell number observed in the mutant.

### Feedback regulation of the insulin pathway on miR-305 expression

Insulin pathway activity regulates ISC growth and proliferation (Amcheslavsky et al. 2009; Biteau et al. 2010; Choi et al. 2011; O’Brien et al. 2011). The ISCs directly respond to the nutritional status of the animal, showing lower insulin signaling via PI3K in nutrient-deprived animals (O’Brien et al. 2011).

**Figure 6.**
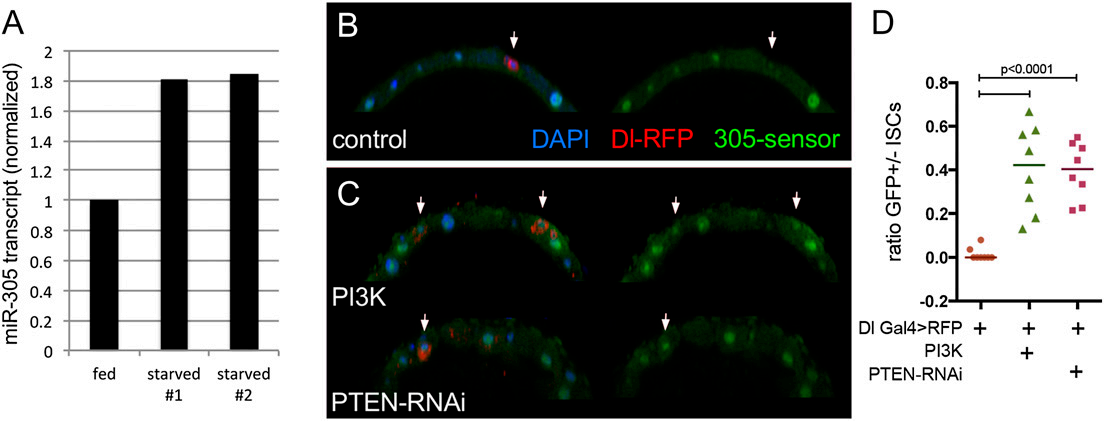
Regulation of miR-305 by the insulin pathway. (*A*) qRT–PCR to measure the unprocessed miR-305 primary transcript in RNA isolated from ISCs by TU tagging. The level of miR-305 primary transcript increased versus the fed control in two independent experiments. Flies were 7 d of age at the time of TU labeling. (*B*,*C*) miR-305 sensor transgene expression. Dl-Gal4 was used to direct UAS-RFP in the ISCs (arrows). (*B*) Control. (*C*) Dl-Gal4 + UAS-RFP + UAS-PI3K or UAS-PTEN^RNAi^ transgenes were used to activate the IIS pathway in the ISCs and were examined at 7 d of age. (*D*) Ratio of Dl^+^ ISCs that showed miR-305 sensor GFP expression versus those without detectable levels of GFP for the experiment in *B* and *C*. ANOVA: *P* < 0.0001 for UAS-PI3K and for UAS-PTEN-RNAi compared with the control.

Given that *miR-305* acts in ISCs to control InR and PI3K levels, we asked whether *miR-305* activity might itself be nutritionally regulated. To monitor the effects of nutrient deprivation on miR-305 levels, we measured the level of *miR-305* primary transcript by quantitative real-time PCR on RNA selectively labeled in ISCs by TU tagging. The 5961GS-Gal4 driver is expressed in ISCs (Biteau et al. 2010). RU486-inducible 5961GS Gal4 was used to drive expression of UAS-UPRT in adult ISCs. RNA was recovered from dissected midguts and assayed for *miR-275–305* primary transcript levels. *miR-275–305* primary transcript increased under nutrient deprivation conditions in two independent trials (Fig. 6A).

In normally fed animals, miR-305 sensor GFP expression in the Dl-expressing ISCs was close to background levels due to the presence of the miRNA (Fig. 6B, arrow), so we could not use the sensor to visualize the effects of increased miRNA expression following nutrient deprivation to reduce IIS activity. However, increasing IIS activity by expressing a UAS-PI3K transgene in the ISCs under Dl-gal4 control increased sensor GFP levels (Fig. 6C). Similarly, increasing IIS pathway activity by expression of a UAS-RNAi transgene to deplete PTEN led to increased sensor levels in the ISCs (Fig. 6C). Increased sensor GFP indicates lower miRNA activity. Thus, increased IIS pathway activity selectively in the ISCs lowers miR-305 activity. Taken together, these experiments provide evidence that *miR-275–305* gene expression in the ISCs is regulated by nutrient signaling through the IIS pathway.

### miR-305 is required for adaptive homeostasis

**Figure 7.**
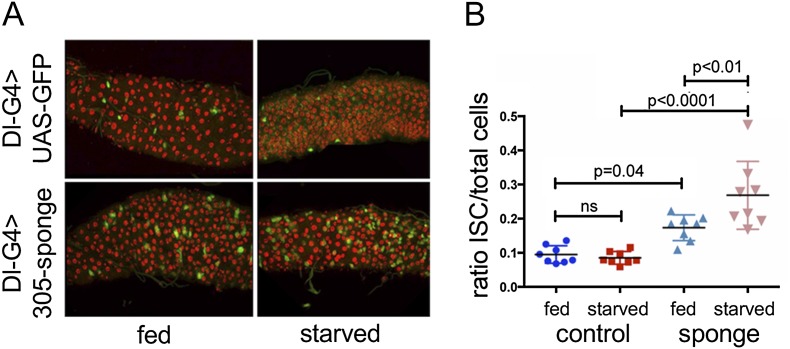
Regulation of miR-305 by the insulin pathway. (*A*) Dl-Gal4 was used to drive expression of the UAS-miR-305 sponge or a control UAS-GFP transgene. Dl-Gal4 was combined with Gal80^ts^. Animals were raised at the permissive temperature to maintain low Gal4 activity, shifted to 29°C at 7 d of age to activate Dl-Gal4 in the ISCs, and aged under fed or starved conditions for 5 d. Representative images are shown. (*B*) Ratio of Dl-Gal4-expressing cells to total cells (*n* = 10 midguts for each genotype and treatment). Horizontal lines show median ± SD. Data were analyzed by ANOVA.

ISC proliferation is sensitive to the nutritional status of the animal (Choi et al. 2011; O’Brien et al. 2011). Feeding induces ISC proliferation through regulation of dILP3 expression in the midgut stem cell niche, providing an adaptive mechanism linking gut growth and ISC self-renewal to nutrient availability (O’Brien et al. 2011). The preceding experiments provided evidence that IIS pathway activity also regulates *miR-305* gene expression in the ISCs. We therefore asked whether regulation of *miR-305* plays a role in adaptive homeostasis of the gut by comparing the effect of nutrient deprivation on ISC number under normal and miR-305-depleted conditions. Nutrient deprivation had no significant effect on the proportion of ISCs identified by Dl-Gal4 expression in the control animals (ANOVA: *P* = 0.98, *n* = 8) (Fig. 7). The proportion of ISCs in nutrient-deprived animals depleted of miR-305 increased compared with the control (ANOVA: *P* < 0.0001) and with the fed miR-305-depleted animals (*P* < 0.01). Thus, the normal balance between the number of progenitor ISCs/EB cells and mature absorptive ECs was perturbed in the miR-305-depleted gut, and this defect was exacerbated following nutrient deprivation. These findings suggest that loss of the miRNA perturbs adaptive homeostasis of the gut by perturbing the mechanism by which nutritional signaling controls stem cell activity.

To address how the balance between the number of ISC progenitor cells and EB cells was perturbed, we counted the number of divisions that produced two identical daughter cells or two different daughter cells. The Gal80ts system was used to allow temporal control of Gal4 activity. Dl-Gal4 was activated in adult ISCs by shifting the animals to 29°C to inactivate Gal80, and the progeny of the next division of the Dl-Gal4-expressing ISCs were examined by labeling with antibody to Dl protein to visualize ISCs and with anti-βGal to visualize Su(H)lacZ expression in EB cells. The daughter cells of the first division were identified as pairs of adjacent cells expressing UAS-dsRed. Divisions producing two Dl^+^ cells or two Dl^−^ βGal^+^ cells represent symmetric division of the ISC progenitor. Asymmetric divisions produced a labeled pair consisting of one ISC and one EB daughter. The difference in the number of symmetric divisions that produced two ISC daughter cells was highly significant when comparing ISCs depleted of miR-305 versus control cells (*P* = 0.0001, Fisher’s exact test) (Table 1). There was no significant difference in the number of divisions producing two EB daughter cells (*P* = 0.13). Thus, the increase in ISC number in the miR-305 mutant appears to result from a change in the proportion of divisions that produce two ISC daughter cells at the expense of divisions producing one stem cell and one EB daughter cell. This effect is exacerbated under conditions of nutrient deprivation.

**Table 1.**
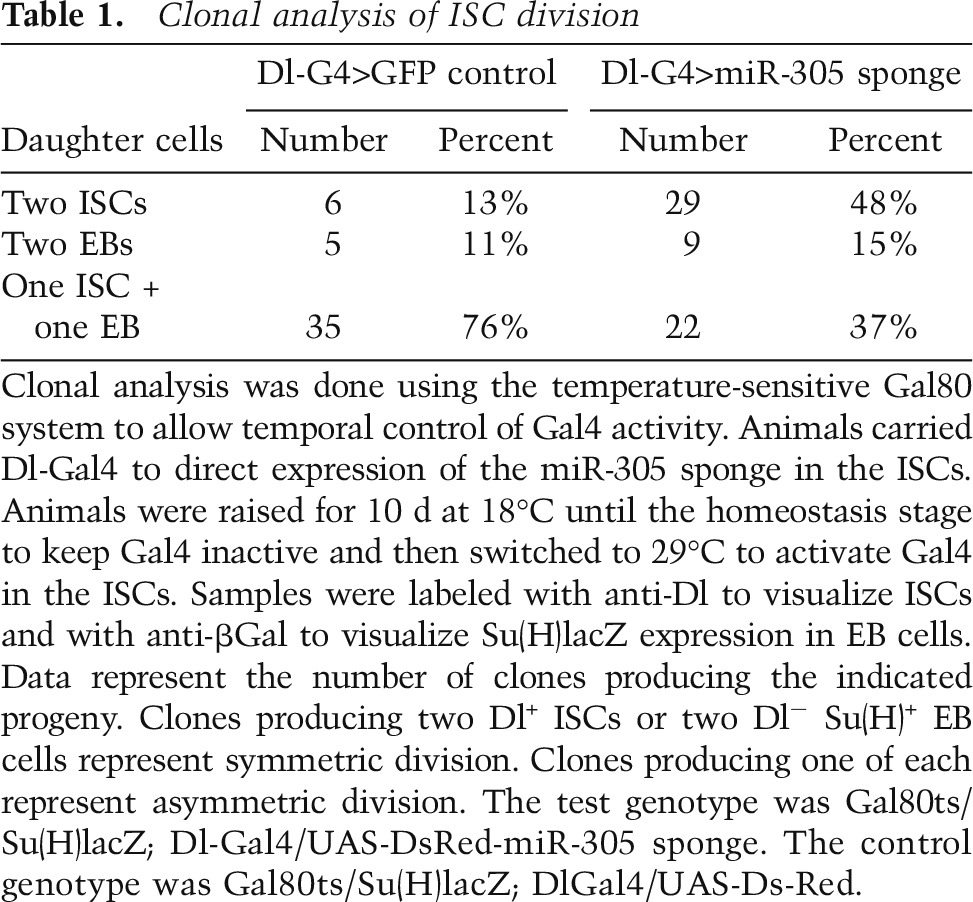
Clonal analysis of ISC division

## Discussion

### Positive feedback: regulation of miR-305 by IIS potentiates responsiveness

The insulin pathway plays a central role in homeostasis of the gut. IIS pathway activity controls the balance between stem cell self-renewal and differentiation. This is regulated in turn by the nutritional status of the animal, linking expansion of the gut stem cell population and gut growth to nutrition, a process termed adaptive homeostasis (O’Brien et al. 2011). Adaptive homeostasis has been linked to nutritional regulation of insulin-like peptides (ILPs) by the stem cell niche, which controls the balance of stem cell proliferation and self-renewal. Our findings implicate miR-305 as a mediator of adaptive homeostasis. miR-305 acts in the ISCs to regulate IIS pathway activity by controlling expression of the ILP receptor (InR) and PI3K. Interestingly, miR-305 expression in the ISCs is nutritionally regulated. Under conditions of reduced nutrient availability, the niche cells produce less ILP. The concomitant increase in miR-305 activity in the ISCs reduces their responsiveness to the niche cell ILP signal. These two effects combine to limit stem cell proliferation and self-renewal when environmental conditions do not support growth and presumably act to ensure a rapid and full response when environmental conditions support growth. The impaired adaptive response to reduced nutrition that was observed in the absence of the miRNA highlights the importance of balancing stimulus and response in this system. miR-305 does not have mammalian orthologs with perfect seed matches. However, hsa-miR-4529-3p shares seven out of eight seed residues with *Drosophila* miR-305 and shares PI3K as a predicted target (Supplemental Fig. S6). Whether this miRNA or other miRNAs with seed similarity to miR-305 play a comparable role in mammalian ISCs remains to be determined.

The homeostatic mechanisms that maintain the gut break down in older flies. With age, the proportion of stem cells increases at the expense of more mature differentiated cells. A dysplastic phenotype that resembles normal gut aging can be induced experimentally by excessive activity of the IIS pathway (Biteau et al. 2010). Our findings provide evidence that the *miR-275–305* mutant leads to elevated IIS activity in the ISCs, even in young animals under good nutritional conditions. This may explain the shortened life span of *miR-275–305* mutants, given that gut dysplasia induced by elevated IIS activity can shorten adult life span (Biteau et al. 2010).

### miR-305 brings Notch activity under IIS control

It is noteworthy that miR-305 acts directly on two pathways that regulate the balance between symmetric and asymmetric ISC division. The Notch pathway plays a key role in the balance between ISC self-renewal and differentiation. ISC differentiation depends on Notch activity (Micchelli and Perrimon 2006; Ohlstein and Spradling 2006). Expression of the Notch ligand Dl is required in the ISCs, and elevated Notch activity is required in the differentiating EB and EC cells (Ohlstein and Spradling 2007; Bardin et al. 2010). However, a careful study of ISC lineages has shown three possible outcomes of ISC division: symmetric divisions producing two ISCs, divisions producing one ISC and one EB daughter cell, and divisions producing no ISCs in which both progeny differentiate (de Navascues et al. 2012). The former leads to expansion of the ISC population, whereas the latter leads to ISC loss. The balance between these outcomes depends on the level of Notch pathway activity, with lower Notch activity favoring ISC pool expansion, and higher Notch activity favoring differentiation (de Navascues et al. 2012). Hairless serves as a negative regulator of Notch signaling and is normally required for ISC maintenance (Bardin et al. 2010). Our findings show that misregulation of Hairless contributes to expansion of the ISC population in the *miR-275–305* mutant. Elevated Hairless expression in the ISCs contributes to an increased frequency of divisions in which both daughter cells adopt ISC identity.

Evidence that IIS regulates the balance between symmetric and asymmetric ISC division is compelling (O’Brien et al. 2011). IIS activity in the EB cells also appears to contribute to the separation of the EB daughter cells from the ISCs, which is required to allow the ISCs to continue proliferating (Choi et al. 2011). Still, no molecular mechanism has been put forward to explain how IIS activity influences whether ISC division produces two ISC daughters versus one ISC and one EB. We propose that regulation of miR-305 expression in the ISCs provides that mechanism. We provided evidence that miR-305 expression in the ISCs is under IIS control. High IIS activity in the ISCs will reduce miR-305 levels, leading to elevated Hairless expression. This, in turn, would lower Notch activity in the ISCs, favoring symmetric division to produce two ISC daughter cells (de Navascues et al. 2012). Conversely, nutrient deprivation reduces IIS activity, leading to higher miR-305 expression and lower Hairless levels. Higher Notch activity then favors divisions that produce an ISC and a differentiating EB daughter cell. Thus, IIS-mediated regulation of miR-305 in the ISCs allows nutritional status to regulate the stem cell pool. Adaptive homeostasis fails in the absence of this miRNA.

## Materials and methods

### *Drosophila* stocks

*Dl-Gal4 P{GawB}*^*NP0677*^ was obtained from *Drosophila* Genetic Resource Center (DGRC), Kyoto; *esg-GFP*^*P01986*^ was from FlyTrap (Buszczak et al. 2007). RNAi lines from Vienna *Drosophila* RNAi Center (VDRC) were Hairless (KK 104341), PI3K (KK 107390), InR (GD 991), and PTEN (GD 35731). RNAi lines from Bloomington Stock Center were *w*^*1118*^, *cuc*^*1*^, *cuc*^*10607*^, *Df(2L)BSC189*, *how-Gal4 P{GawB}*^*24B*^, and *tub-Gal80*^*ts*^. *hsFlp; tub-Gal4, UAS-GFP; FRT40A tub-Gal80*^*ts*^ was provided by M. Milán. *esg-Gal4*, *Su(H)GBE-Gal4 5961GS Gal4*, and *UAS-H* were provided by J. de Navascués, S.X. Hou, H. Jasper, and J.F. de Celis.

### Generation of mutants and other transgenic flies

Gene targeting by ends-out homologous recombination using the pW25 vector was carried out as described (Chen et al. 2011). Deletion of *miR-275* and *miR-305* hairpin DNA was verified by PCR on genomic DNA and by TaqMan miRNA quantitative PCR (qPCR). UAS-miR-275 and UAS-miR-305 were made by amplifying 174 and 162 base pairs (bp) of genomic DNA containing the miRNA hairpins (primers: UAS-miR-275-F, 5′-TGTAGCGGCCGCATGATGTTCCCCCGACTGTA-3′; UAS-miR-275-R, 5′-GATCCTCTAGATGCGAGCATTTCGCTTATTT-3′; UAS-miR-305-F, 5′-TGTAGCGGCCGCCGCCAGAAATCCCATGTGTA-3′; and UAS-miR-305-R, 5′-AGATCCTCTAGACTTGTATCGGTCGCTTTCGT-3′). The amplified miRNA hairpin DNAs were cloned into the NotI and XbaI sites of pUAST-dsRED (Brennecke et al. 2005). The *miR-305* GFP sensor was made by cloning two copies of the sequence complementary to *miR-305* into the 3′ UTR of GFP in pTubulin-GFP-SV40 (Brennecke et al. 2003). The miR-275 and *miR-305* sponges were made by cloning 10 copies of a complementary sequence to the miRNA (with a mismatch at base 10) into the 3′ UTR of pUAST-dsRED.

### Immunostaining and EdU incorporation

Adult midguts were dissected in PBS and fixed for 30 min at room temperature in 4% (v/v) paraformaldehyde in PEM (0.1 M PIPES, 2 mM EGTA, 1 mM MgSO_4_). Triton-X and sodium deoxycholate were added to the fixative to a final concentration of 0.1% (w/v). Tissues were washed four times in PBS and incubated overnight at 4°C with primary antibodies, washed again with PBS, and incubated for 2 h with secondary antibodies. Samples were mounted in VectaShield before imaging. The antibodies used were rabbit anti-pAkt-505 (1:100; Cell Signaling), mouse anti-Dl (1:30; Developmental Studies Hybridoma Bank), Alexa 555 anti-mouse, Alexa 647 anti-mouse, and Alexa 488 anti-rabbit IgG*.* For proliferation assays, midguts were incubated in 5 µM EdU for 30 min immediately after dissection according to the manufacturer’s instructions (Invitrogen). Nuclei were visualized with DAPI.

### Clonal analysis

MARCM clones were induced in 2- to 3-d-old adult flies by a 30-min, 37°C heat shock. The genotype used was *hsFlp; tub-Gal4, UAS-GFP; FRT40A tubGal80/FRT40A miR-275-305 KO*.

### Luciferase reporter assays

A Tubulin promoter–miR-305 expression construct was prepared as described (Brennecke et al. 2003). S2 cells were transfected in 24-well plates with 250 ng of tubulin-miR-305 plasmid or with the empty tubulin–promoter vector as a control. Cells were cotransfected with 50 ng of firefly luciferase reporter vector carrying the SV40 3′ UTR as a control or with the target 3′ UTR or a mutant version of the UTR reporter in which the target site sequences were altered as indicated in Figure 5A. Cells were also cotransfected with 50 ng of Renilla luciferase DNA as a transfection control. Samples were harvested 60 h after transfection, and dual luciferase assays were performed (Promega). Four biological replicates were performed for each experiment.

### TU tagging of RNA in adult ISCs

For Figure 5C, Dl-Gal4, Tub-Gal80^ts^ UAS-UPRT flies were raised until 5 d of age at 18°C and shifted for 2 d to 29°C to induce Gal4 activity in the ISCs. After 48 h, flies were transferred to empty vials for 12 h and then to 4-TU-containing food. After 12 h on TU medium, midguts were dissected from 30 flies. RNA was extracted using Trizol, and tagged RNA was purified as described (Miller et al. 2009). For Figure 6A, ISC-specific expression of UAS-UPRT was under the control of the RU486-inducible 5961GS Gal4 (Biteau et al. 2010). Freshly eclosed adults were collected and aged in groups of 30 in normal food vials for 5 d at 25**°**C. Flies were transferred to food containing RU486 to induce Gal4 activity in the ISCs (and reared at 29°C to increase Gal4 activity). One group of flies was maintained on normal food vials containing RU486 (“fed”). The other was transferred to vials containing 1% agarose containing RU486 (“starved”). After 48 h, both groups were processed as described above.

### qPCR

TaqMan miRNA assay reagents to mature *miR-275* or *miR-305* by qPCR were from Applied Biosystems. miRNA levels were normalized to U14 or snoR442 reference RNAs. For mRNA qRT–PCR, first strand cDNA synthesis used random hexamer primers and SuperScript RT-III (Invitrogen). Measurements were normalized to actin42A as a control. Primer sequences for PCR amplification of the primary miR-275–305 transcript were Pri305F (5′-CAAATCGCCTCATATTGAGTGT-3′) and Pri305R (5′-TCCCATGTCTATTGTACTTCATCA-3′). Other primer sequences are available on request.

### Longevity/nutrient deprivation assays

Adult flies were collected at eclosion; males and virgin females were aged separately in groups of ∼20 flies per vial. The food recipe was as follows: 5.8 g of cornmeal, 5.1 g of dextrose, 2.4 g of brewer’s yeast, 0.8 g of agar, and 100 mL of distilled water. Nipagen (10% in ethanol) was added to 0.3% after autoclaving. Nutrient deprivation was done in vials containing 1% agarose. For experiments requiring several days of deprivation, 1% agarose and 1% sucrose were used, as described (O’Brien et al. 2011). Flies were raised at 25°C and 65% humidity on a 12-h light/dark cycle unless otherwise indicated. Surviving flies were counted daily and transferred to fresh vials.

### Image analysis

Stacks of confocal optical sections were collected, and maximum projections were prepared using a Zeiss LSM510 microscope. Images were adjusted for brightness and contrast. Cell numbers were counted in a standardized area comprising 300 × 350 µm in the posterior side of midgut region P3.
